# Editorial: Principles and Challenges of Fundamental Methods in Veterinary Epidemiology and Economics

**DOI:** 10.3389/fvets.2021.705980

**Published:** 2021-06-14

**Authors:** Salome Dürr, Victoria J. Brookes, Andres M. Perez

**Affiliations:** ^1^Veterinary Public Health Institute, Vetsuisse Faculty, University of Bern, Bern, Switzerland; ^2^Faculty of Science, School of Animal and Veterinary Sciences, Charles Sturt University, Wagga Wagga, NSW, Australia; ^3^Graham Centre for Agricultural Innovation (NSW Department of Primary Industries and Charles Sturt University), Wagga Wagga, NSW, Australia; ^4^College of Veterinary Medicine, University of Minnesota, St Paul, MN, United States

**Keywords:** method, guideline, introduction, discipline, epidemiology, economics

The discipline of veterinary epidemiology focuses on the investigation of the dynamics, frequency, and determinants of diseases in populations of veterinary interest. Epidemiological methods are continuously changing, as new tools and techniques become available, often borrowed from other disciplines and adapted to veterinary science objectives. Therefore, there is a need for epidemiologists to become acquainted with both existing and emerging methods to take advantage of all available approaches to support the prevention and control of disease in animal populations.

Internationally recognised epidemiologists have been invited to contribute to this Research Topic with articles that cover existing and emerging areas of epidemiological research with a focus on their use in veterinary research. Whilst some methods presented here have been used for decades, others have evolved relatively recently. To promote their use by the broad community of veterinary epidemiologists worldwide, the articles have been written to introduce methodologies to researchers who are relatively new to the respective topic. The principles, advantages, challenges and limitations, as well as perspectives on how these methods will evolve given their use in a veterinary epidemiology context, are discussed in each article, so that they can be used as guidelines for application. To support the practical use of the methods presented in the articles, code and example data have been provided where possible.

Articles have been grouped into three sections: (1) assessing the literature, collecting data, and measuring disease, (2) identifying epidemiological associations and exploring disease patterns, and (3) modelling disease and estimating its economic impact.

Methods to assess the literature, collect data, and measure the impact of disease are fundamental aspects of veterinary epidemiology and economics. Six articles are allocated to this area in this Research Topic. Sargeant and O'Connor provided an overview on scoping review, systematic reviews, and meta-analysis, and their application in veterinary science. They highlight that these methods are becoming increasingly popular for both researchers and practitioners; understanding the distinction between review types is important to be fit for purpose. For example, whilst scoping reviews might map the broad knowledge around an area of veterinary science, a systematic review might be more useful to identify literature relevant to a more specific area. Finally, meta-analyses quantitatively combine the results from multiple studies that have been identified by systematic reviews.  Hu et al. describe a type of meta-analysis, the Bayesian network meta-analysis, in more detail and illustrate the procedures and stepwise workflow, including a description of how to informatively present the results in ranking plots and treatment risk posterior distribution plots.  Brennan et al. present another approach for systematic evidence synthesis: Critically Appraised Topics (CATs). This method uses the same principles as a systematic review, but is aimed at addressing a clinically-based question from a veterinary professional to support evidence-based clinical practice. The authors illustrate five steps of CATs based on an example, and emphasise the clinical relevance and practicalities. Stevenson provides a guide to a fundamental concept in epidemiology: sample size estimation. Justification of the number of subjects enrolled into a study and how this has been calculated are a core requirement of any epidemiological study, to demonstrate sufficient power whilst balancing resources such as time and sampling cost. Animals are typically aggregated into groups leading to a lack of independence of observations, and approaches are discussed to overcome this issue. The article by Degeling and Rock presents principles of qualitative research for One Health projects. They highlight the potential of collaborative projects between qualitative researchers and veterinary epidemiologists by emphasizing how qualitative research can contribute to better interpretation of findings in different cultural, economic, historical, and social contexts. As such, qualitative methods support epidemiological researchers to develop policy so that it can be implemented with a more politically and socio-culturally meaningful approach. Several methods, such as interviews, participant observations and working with groups are presented, as well as useful ways to analyze such data. Alders et al. contribute an article on participatory epidemiology (PE) in veterinary science, a method that has evolved to embrace knowledge, experience, and motivations of relevant stakeholders, such as animal caretakers and owners, for identification and assessment of animal disease problems. The review article describes the evolution of PE, its philosophy and principles for effective application, and the importance of data triangulation and gender- and minority-sensitive approaches.

Identifying epidemiological associations and exploring spatial and temporal disease patterns are key domains for veterinary epidemiology and economics. Five articles were collated in this section. Kratzer et al. introduce the readers to Bayesian network (BN) modelling, described as a flexible analytical framework for complex epidemiological datasets. In veterinary science, we are often confronted with datasets containing interdependent variables, which challenge classical uni- and multi-variable regression models used for risk factor analyses. BN modelling is an approach which aims to overcome these issues, and untangle direct and indirect relationships between variables. BN modelling is described and applied using a stepwise approach to a veterinary dataset, and results are compared to a classical regression approach. Kanankege et al. illustrate an overview of spatiotemporal visualisation and analytical tools (SATs) in population-level eco-epidemiological research, and present them in a framework for choosing the appropriate method for a specific research question and dataset. Following a stepwise process based on six research questions, researchers are directed to select a suitable SAT, belonging to one of four categories: (1) visualisation and descriptive analysis, (2) spatial or spatiotemporal dependence and pattern recognition, and (3) spatial smoothing and interpolation and (4) geographic correlation studies for testing inferences in spatial dependent datasets. Escobar presents an article on ecological niche modelling. He highlights the increasing importance of distributional ecology in the field of epidemiology, since the biotic interactions between pathogens and hosts are crucial for infectious disease diversity, distribution and maintenance. Development of interdisciplinary research methods that bring together ecology and epidemiology, such as ecological niche modelling, will improve predictions for infectious disease abundance. Ward et al. present and discuss methods used to analyse time-series data in veterinary science, focusing on ARIMA (Autoregressive Integrated Moving Average) models. Time-series datasets are relatively common in animal disease monitoring and surveillance systems, but can also originate from animal production or welfare datasets that are increasingly available in modern animal production systems. Although many datasets in veterinary science could be analyzed using this method, it was found to be relatively rarely used in this field. The stepwise instructions and code provide veterinary epidemiologists with foundation skills in this method. Alkhamis et al. introduced the use of phylodynamic methods as a recently evolved method for emerging and endemic animal viral disease surveillance. Phylodynamic methods offer the possibility to integrate spatio-temporal epidemiology and evolutionary dynamics into one analysis framework by using single Bayesian statistical techniques on the phylogeny of viruses in populations. The practical steps required to perform phylodynamic analyses (sequence preparation, preliminary phylogenetic analysis, selecting and running phylodynamic models, and visualisation of the model outcomes) are presented, and the robustness of different models is tested. Challenges for integrating phylodynamic methods in routine animal disease surveillance activities are also discussed.

The use of infectious disease modelling has been increasing over previous decades and nowadays comprises diverse methodologies. Three articles describe different methods, and a fourth focuses on the estimation of the economic impact of infectious diseases. Brzoska et al. present a relatively new method called stochastic block modelling (SBM) to unravel complex networks such as those existing in animal trade. SBM splits such networks into smaller units of nodes (for example, farms) that show similar network properties. The method was shown to perform better for informing trade restrictions to control diseases, compared to the more established community detection method or trade restrictions based on geographical boundaries. Kinsley et al. also illustrate novel network methodologies, namely multilayer and multiplex networks. Multilayer networks account for different modes of spread of pathogens between hosts, and therefore, consider multiple layers of contacts resulting in multiplex networks. Also, different types of hosts can harbor the same pathogens within one ecosystem, which can be illustrated by interconnected multilayer networks. In this article, these techniques are reviewed, and applied to an example to demonstrate how these models can capture disease dynamics in complex host-pathogen systems. Kirkeby et al. present an introduction to mechanistic modelling of disease transmission, focusing on individual-based models that allow inclusion of heterogeneity between individual epidemiological units. The article illustrates and describes important steps before, during and after model programming. Model verification, validation, convergence analysis, and sensitivity analysis are described and discussed, and examples provided for each of these steps. Gethmann et al. present gross margin analysis (GMA) to estimate the economic impact of an infectious disease outbreak. They implement GMA within the Excel add-in @Risk (Palisade), an easy-to-use, commercial tool for stochastic simulations. Within their @Risk model, direct costs (for example, production losses, animal deaths, veterinary treatment) and indirect costs (for example, surveillance, measures for animal export, disease control, vector monitoring, and administration) can be individually entered. Such tools can therefore be suitable for economic impact estimation, thus using GMA for broad applications in veterinary economic.

In summary, this Research Topic provides an overview of existing, emergent and novel methods with applications in veterinary epidemiology. We expect that this contribution will provide the veterinary epidemiology community with resources to improve their knowledge on existing and novel methods, thus supporting the prevention, surveillance and control of diseases that impact both human and animal health.

## Author Contributions

All the authors were involved in writing the article and agreed to the final version of this editorial.

## Conflict of Interest

The authors declare that the research was conducted in the absence of any commercial or financial relationships that could be construed as a potential conflict of interest.

